# Effect of a longpass filter on myopic defocus-induced changes in axial length and choroid thickness

**DOI:** 10.3389/fnins.2026.1783536

**Published:** 2026-05-15

**Authors:** Xue Li, Chenyao Liu, Weilin Wu, Daniel P. Spiegel, Björn Drobe, Hao Chen, Jinhua Bao

**Affiliations:** 1 National Engineering Research Center of Ophthalmology and Optometry, Eye Hospital, Wenzhou Medical University, Wenzhou, China; 2 R&D, EssilorLuxottica, Singapore, Singapore

**Keywords:** axial length, blue light, chorodal thickness, emmetropization, myopia defocus

## Abstract

**Purpose:**

To investigate the short-term effect of a longpass filter on myopic defocus-induced changes in axial length (AL) and choroidal thickness (ChT).

**Methods:**

This study included 23 young myopic adults. AL and ChT were measured in both eyes before and after 60 min of +5 D myopic defocus induced in the right eye with either a longpass filter (>495 nm) or a neutral density (ND) filter of equal transmittance. The same ND filter was used for both the left eye and the control eye. The pupil diameter of the left eye was measured before and 10 min after each test session. Illumination was maintained at 100 lux.

**Results:**

After 60 min of monocular defocusing with the ND filter, a significant reduction in AL (mean±SE) was observed in the right eye (−8.20 ± 3.01 μm; *t* = −2.72; *p* = 0.01). However, no significant change was noted in ChT (−0.73 ± 1.78 μm; *t* = −0.41; *p* = 0.69). With the longpass filter, no significant change in AL was observed (−1.11 ± 4.27 μm, t = −0.26, *p* = 0.80), but a significant reduction in ChT was observed (−4.56 ± 1.73 μm, *t* = −2.63, *p* = 0.02). Pupil size did not significantly differ between the two filters (*F* = 0.004, *p* = 0.95).

**Conclusion:**

Blocking short wavelengths inhibited the acute axial shortening response to myopic defocus, suggesting a modulatory role of short wavelengths in refractive development under the present experimental conditions.

## Introduction

In recent decades, the prevalence of myopia has been increasing globally. Outdoor activities can help control or even reduce the incidence of myopia (He et al., 2015, 2022; Wu et al., 2013; Jin et al., 2015). Outdoor activities may help control myopia because of their association with high illuminance levels (Wu et al., 2018), reduced peripheral defocus (Flitcroft, 2012), circadian rhythms (Stone et al., 2013), high spatial frequency (Flitcroft et al., 2020) and a high chromatic spectrum (Xu et al., 2023). Animal studies have shown a correlation between specific light spectra and myopia (Flitcroft, 2012; Flitcroft et al., 2020; Lingham et al., 2020). A study on chicks revealed that the process of emmetropization can be triggered by signals from longitudinal chromatic aberration (Rucker and Wallman, 2009). However, narrow-band lighting cannot maintain emmetropia (Norton et al., 2021; She et al., 2023) and different monochromatic light wavelengths have different effects on refractive development and eye growth in animals. For example, chickens (Rucker and Wallman, 2009; Seidemann and Schaeffel, 2002), guinea pigs (Long et al., 2009) and fish (Timucin et al., 2016) became myopic when exposed to medium- or long-wavelength light but showed more hyperopic tendencies under short-wavelength (blue) light exposure. However, rhesus monkeys (Smith et al., 2015) and tree shrews (Gawne et al., 2017; Ward et al., 2018) do not exhibit these effects; instead, they become hyperopic under long-wavelength light exposure. Therefore, the relationship between monochromatic light and refractive development remains controversial.

While clinical studies on chromatic light and myopia are few in number, emerging evidence points to a significant role of violet light. Retrospective data suggest that blocking violet light (360–400 nm) may lead to greater axial elongation than allowing its transmission, suggesting a protective role of violet light (Torii et al., 2017a,b). Experimental studies further revealed complex wavelength-specific effects: short-term exposure to narrow-band blue light (460 nm) reduced axial elongation, whereas red or green light increased axial elongation (Thakur et al., 2021). Notably, despite the potential of red light to promote elongation in some settings, low-intensity red light therapy has shown efficacy in controlling childhood myopia, presenting an intriguing contradiction (Jiang et al., 2022). Collectively, these findings highlight that chromatic light influences refractive development, yet its precise mechanisms and its interplay with established optical interventions are unclear.

Considering that short-wavelength light exposure is crucial for circadian rhythms and necessary for eye growth regulation, research exploring the combination of chromatic cues with defocus-based interventions is underway. For instance, narrowband blue light demonstrated a complementary protective effect when combined with dual-focus lenses in chicks (Chun et al., 2023). Conversely, another study revealed that blue light itself inhibited ocular elongation, independent of hyperopic defocus, unlike longer wavelengths (Thakur et al., 2021). These animal studies point to a promising yet complex interaction, underscoring the need to clarify how the absence of specific short wavelengths, combined with myopic defocus, affects ocular parameters such as axial length and choroidal thickness in humans—which is the primary aim of the present study.

Short-wavelength (blue) light is crucial for circadian rhythm regulation, and the signalling pathways involved significantly overlap with those controlling eye growth (Stone et al., 2013). Therefore, the aim of this study was to investigate the role of short-wavelength light exposure in myopia control by examining how its absence, when combined with myopic defocus, affects axial length and choroidal thickness.

## Methods

Twenty-three myopic university students, aged between 19 and 30 years (mean ± SD, 24.6 ± 2.3 years), were recruited, including sixteen females. Before the start of the experiment, all participants underwent a comprehensive ocular examination to confirm good ocular health, normal binocular vision, and a normal refractive status. None of the participants exhibited any ocular or systemic disease. Their spherical equivalent refraction (SER), obtained from noncycloplegic subjective refraction, ranged from −1.75 D to −5.00 D. None of the participants exhibited cylindrical refraction exceeding 1.00 D or anisometropia greater than 1.50 D. Best-corrected visual acuity (BCVA) was 0.00 logMAR or better for all participants. This study adhered to the tenets of the Declaration of Helsinki and received approval from the Ethics Committee of the Eye Hospital of Wenzhou Medical University (no. KYK [2017]31). Before participation, all participants provided written informed consent after receiving a detailed explanation of the nature and possible consequences of the study.

### Procedure

Two filters were used: a longpass filter (GG-495; longpass filter; Edmund Optics, Singapore, Singapore), which blocked wavelengths below 495 nm and served as the experimental instrument, and an equally transmissive neutral density (ND) filter (absorptive ND filter; Edmund Optics, Singapore), which served as the control. The experiment was conducted over 2 days, with one filter tested at the same time each day, and at least 24 h between each testing. All measurements were performed between 9 a.m. and 3 p.m. to minimize the potential effects of diurnal variations on axial length and choroidal thickness (Carpena-Torres et al., 2023). Participants were instructed to refrain from consuming beverages containing caffeine or ethanol on examination days, as these substances have been shown to impact choroidal thickness (Chakraborty et al., 2023; Nickla et al., 2023). Room illuminance was maintained at 100 lux throughout the protocol.

**Figure 1:**
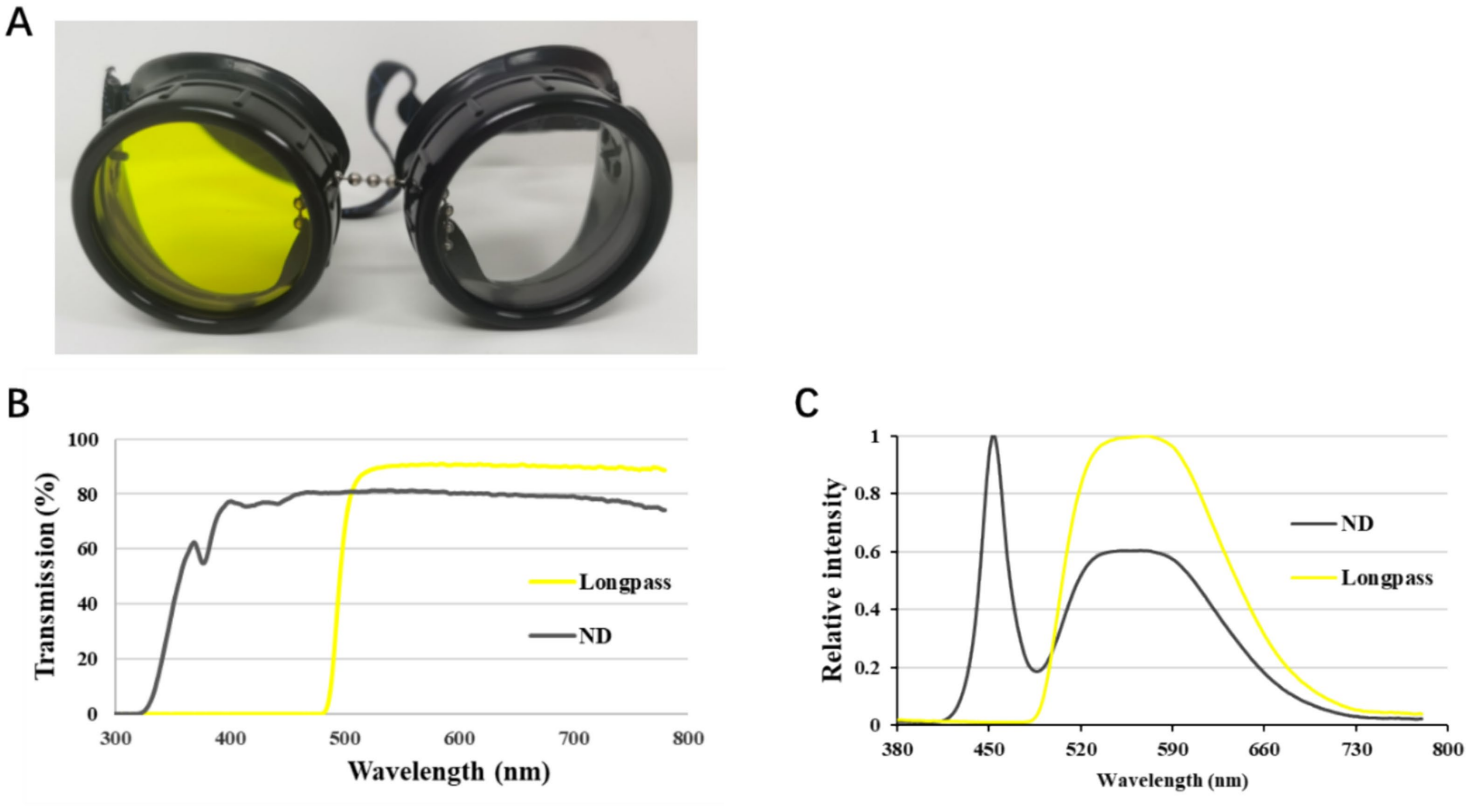
Goggles with an ND filter (**A**, right) and a longpass filter (**A**, left). Both lenses have a diameter of 50 mm. The transmission spectrum of each filter was measured using a spectrophotometer (U4100, Hitachi, Tokyo, Japan) **(B)**, and the spectral profiles at eye level were measured using an MK350N Spectrometer (UPRtek, Taiwan) **(C)**.

During the measurements, participants wore single-vision soft contact lenses (Bausch+Lomb, Ireland) for full correction in both eyes. Goggles were used to manipulate the visual condition (Figure 1A); a + 5.00 D lens and either a longpass filter or an ND filter were placed in front of the right eye, while the same ND filter and a Plano lens were placed in front of the left eye. The ND and longpass filters had visible light transmittances of 81 and 84%, respectively, as measured by a spectrophotometer (U4100, Hitachi, Tokyo, Japan). The spectrum at eye level was measured by an MK350N Spectrometer (UPRtek, Taiwan) (Figure 1).

**Figure 2:**
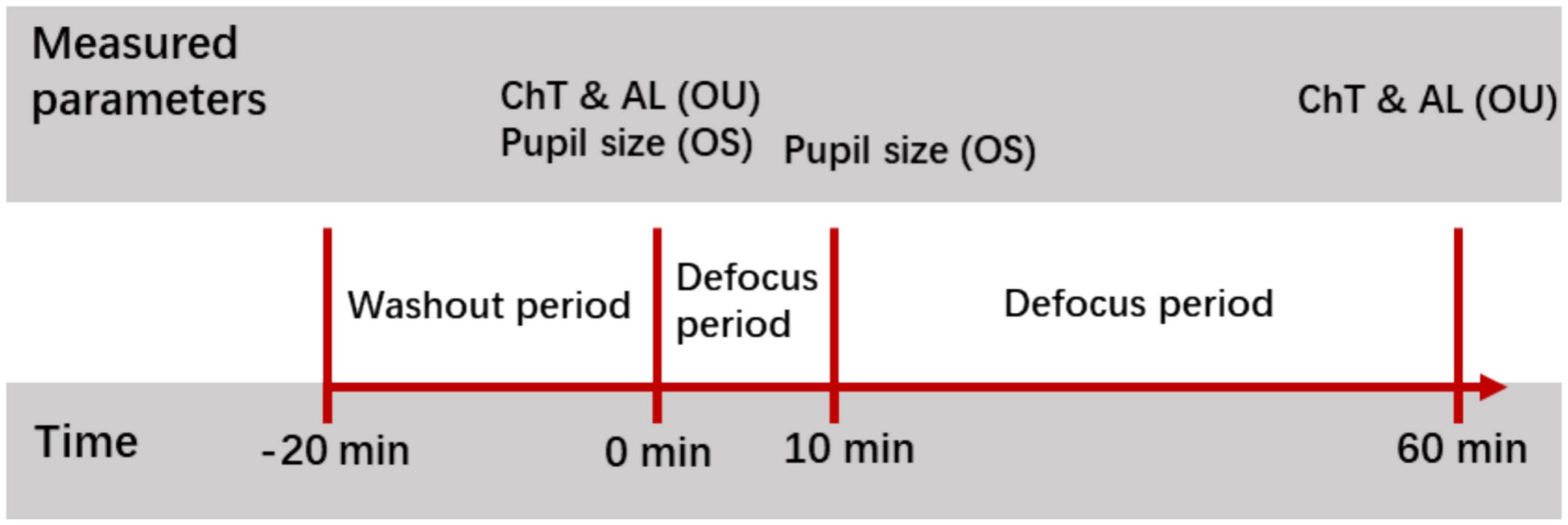
Flowchart of the experimental session. AL, axial length; ChT, choroidal thickness.

The sequence of test sessions with the two filters was randomized for each participant. Participants watched a 20-min documentary series (“The Blue Planet”) on a 55-inch television (LG, 47LW5500-CA) positioned 5 metres away to minimize any potential influence on ocular parameters from previous visual tasks (Torii et al., 2022). Baseline measurements of axial length and choroidal thickness were then obtained. Afterwards, a + 5D lens and one of the filters were introduced in front of the right eye, while the left eye had the ND filter and a Plano lens; participants continued viewing the documentary film for another 60 min. Immediately after the viewing period, axial length and choroidal thickness in both eyes were measured again (Figure 2). Additionally, the pupil size of the left eye was measured before and 10 min after each test session using a GrandSeiko autorefractometer (WAM-5500; Grand Seiko Co., Ltd., Japan).

### Ocular measurements

Measurements were obtained while the participants were wearing soft contact lenses. Choroid images were captured using spectral domain optical coherence tomography (SD-OCT, HRA + OCT, Heidelberg Engineering, Heidelberg, Germany) in enhanced depth imaging (EDI) mode, which provides superior image quality and enables better visualization of the choroidal structure. The measurements comprised an average of 100 B-scans with a mean quality index exceeding 30 dB. The “follow-up” mode was used to ensure consistent positioning on the fundus, and the choroid was measured three times. Custom-written software was used to analyse and segment all the images after correcting for scan width (Liu et al., 2014). The thinnest part of the macula in each image was identified as the location of the fovea. Subfoveal ChT was defined as the distance between the outer surface of the retinal pigment epithelium (RPE) and the inner surface of the choroscleral interface beneath the fovea. Two experienced staff members independently reviewed all the OCT images for each participant and made manual corrections if necessary. The three measurements that were performed by the two staff members were averaged, and the images were reanalysed if the difference was greater than 10 μm.

AL was measured using a noncontact optical biometer (IOLMaster; Carl Zeiss Meditec, La Jolla, CA, USA). Five valid scans (SNR > 2.0) were finally retained and averaged for each time point.

### Statistical analysis

Statistical analyses were performed using SPSS 26 (IBM, Armonk, NY). Changes in ChT and AL were reported as differences between the defocused eye (right eye) and the control eye (left eye). All data are presented as the mean ± standard error. One sample t test was conducted to examine changes in AL and ChT after myopic defocus with any filter. Paired t tests were employed to compare differences in AL, ChT, and pupil size between the ND filter (control condition) and the longpass filter (longpass condition). A *p* value <0.05 indicated statistical significance.

## Results

Of the 23 participants, one participant did not undergo measurement; thus, 22 participants were included in the data analysis (15 females and 7 males). No significant differences were observed in SER, AL, ChT, or BCVA between the right and left eyes at baseline (all *p* > 0.05; Table 1).

**Table 1:** Baseline ocular parameters of the 22 participants.

Variables	Right eye	Left eye	*p* values
SER (D)	−3.38 ± 0.19	−3.20 ± 0.19	0.10
AL (mm)	25.28 ± 0.18	25.18 ± 0.19	0.07
ChT (μm)	269.42 ± 12.10	266.38 ± 13.63	0.67
BCVA, LogMAR	−0.09 ± 0.02	−0.10 ± 0.01	0.43

SER, spherical equivalent refraction; AL, axial length; ChT, choroidal thickness; BCVA, best-corrected visual acuity. The data are presented as means ± SE.

### Changes in AL

**Figure 3:**
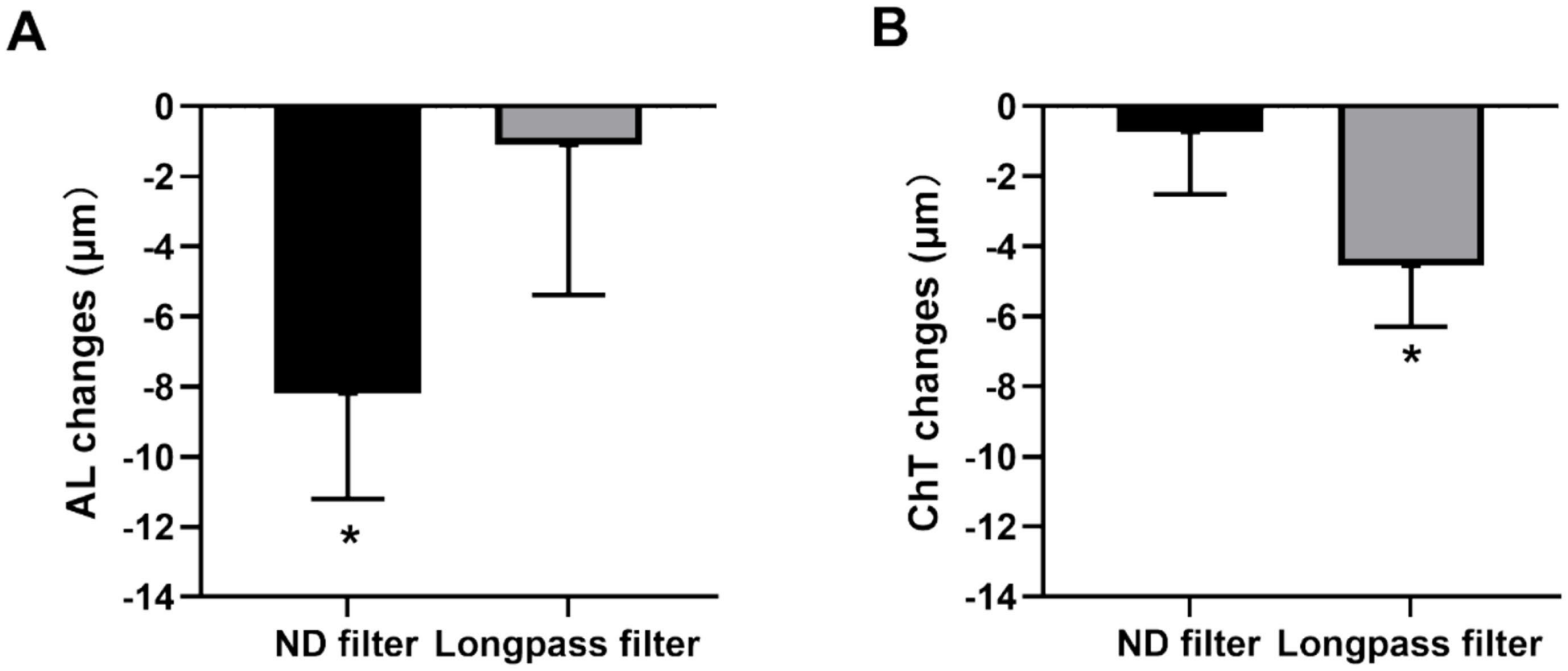
Changes in AL **(A)** and ChT **(B)** after 60 min of defocusing with the ND filter or the longpass filter. AL, axial length; ChT, choroidal thickness; ND, neutral density. The error bars represent the standard error of the mean. *p < 0.05.

After 60 min of myopic defocus, a significant reduction in AL of 8.20 ± 3.01 μm (mean ± SE; *t* = −2.72, *p* = 0.01) was observed with the ND filter (Figure 3A). However, no significant change in AL was observed with the longpass filter (−1.11 ± 4.27 μm, mean ± SE; *t* = −0.26, *p* = 0.80; Figure 3A).

### Changes in the ChT

One participant was excluded from the ChT analysis because the image quality was less than 30 dB. After 60 min of monocular defocus, the change in ChT exhibited an opposite trend to that in AL with both filters (Figure 3B). With the ND filter, no significant changes in ChT were observed (−0.73 ± 0.78 μm, mean ± SE; *t* = −0.41; *p* = 0.69). Conversely, the change in ChT was significant with the longpass filter (4.56 ± 1.73 μm, mean ± SE; *t* = −2.63, *p* = 0.02; Figure 3B).

### Changes in pupil size

Two participants were excluded from pupil data analysis because of excessive blinking. The changes in pupil diameter with the two filters are presented in Table 2. Time had a significant main effect on changes in pupil size (F_1,19_ = 20.72, *p* < 0.001); however, there was no significant interaction effect between filter and time (F_1,19_ = 0.15, *p* = 0.70).

**Table 2:** Pupil diameter (mm) before and after 10 min of defocus with each filter (*n* = 20; Data, mean ± SE).

Condition	ND filter	Longpass filter	P^a^
Before defocus	5.86 ± 0.17 mm	5.87 ± 0.13 mm	0.95
After defocus	5.64 ± 0.16 mm	5.68 ± 0.15 mm	
P^b^	<0.001		

a Refers to the difference in pupil size between the ND and longpass filters.

b Refers to the changes in pupil size before and after 10 min of defocus.

## Discussion

This study investigated how myopic defocus affects AL and ChT with either the longpass filter (blocks all blue light below 495 nm) or an equally transmissive neutral ND filter as the control. Under the neutral filter with a + 5D lens, AL in the defocused eye significantly decreased, while ChT did not change significantly. Conversely, with the longpass filter and a + 5D lens, AL did not change significantly in the defocused eyes, but ChT significantly decreased.

The observed axial shortening (micrometer-scale over 60 min) likely reflects transient physiological changes, rather than long-term structural remodeling of the eye. Therefore, these short-term measurements should not be directly equated with clinically meaningful myopia control efficacy. However, they provide valid mechanistic insight into how the spectral composition of light influences the eye’s acute response to myopic defocus.

Previous animal studies have indicated that monochromatic light can influence refractive development by inducing wavelength-specific defocus. Longitudinal chromatic aberration (LCA), whereby long-wavelength (“red”) light is focused behind short-wavelength (“blue”) light, provides a potential cue for detecting refractive error. However, different species exhibit varying compensatory responses to such monochromatic defocus. The underlying mechanism for these interspecies differences remains unclear but may involve variations in circadian rhythm and associated hormonal pathways and differences in their responses to light intensity and spectral bandwidth. Synchronization between the retinal circadian clock and the central pacemaker in the suprachiasmatic nucleus, along with the balanced secretion of key signalling molecules such as dopamine and melatonin, plays a crucial role in refractive development (Nickla, 2013; Chakraborty et al., 2018). Interspecies variations in these circadian parameters could therefore lead to divergent responses to optical defocus or light-based interventions. It is suggested that broader spectral bandwidths provide more reliable cues for emmetropization (Xu et al., 2023; Hu et al., 2022), whereas narrow-band blue light may generate signals that are easily confounded with defocus. To specifically investigate the role of blue light in emmetropization while avoiding the confounding effects of narrow-band stimulation, a longpass filter was employed to block short-wavelength light (<495 nm) rather than monochromatic blue light.

In the present study, AL shortening caused by +5 D myopic defocus was effectively suppressed with the use of a longpass filter that fully blocks blue light. This finding is consistent with those of a previous animal study conducted by Xu et al. (2023), who demonstrated that blocking blue light (<400 nm) failed to inhibit axial growth elongation in tree shrews compared with natural light. On the other hand, Mori et al. reported that wearing violet light permission glasses for 2 years reduced axial elongation by 21% in myopic children aged 6–12 years compared with those in the placebo glasses group; this reduction was observed only when the children spent less than 180 min on near-work and only among those who had never worn eyeglasses before (Mori et al., 2021). Furthermore, the addition of violet light was found to be effective against myopia progression in a small sample of myopic children aged 8 to 10 years over a period of 6 months (Torii et al., 2022). However, only a few clinical trials have explored the use of short-wavelength light for myopia control, and these studies had short follow-up times, small sample sizes and limited efficacy. These results collectively suggest a potentially important role of short-wavelength light exposure in myopia control. The underlying mechanism involves violet light within a specific band (approximately 360–430 nm) penetrating the ocular media and activating specialized retinal photoreceptor pathways (e.g., ipRGCs and S-cones), thereby triggering a downstream neurovascular signalling cascade that ultimately modulates excessive axial elongation (Gawne et al., 2017; Patterson et al., 2022).

In addition to LCA, animal studies have recently shown that blue light exposure may regulate myopia by increasing the blood supply to the choroid and increasing choroidal thickness in a time-dependent manner (Wang et al., 2023). Short-wavelength (blue light) exposure may also be involved in the regulation of myopia by promoting the secretion of dopamine (Carpena-Torres et al., 2023). Moreover, Chakraborty et al. reported that melanopsin stimulation with blue light exaggerated the ocular response to hyperopic defocus but had no effect on myopic defocus, suggesting a potential interaction between myopiagenic hyperopic defocus and the ipRGC system (Chakraborty et al., 2023). There was a more complex interaction between illuminance, defocus, and time of day in the study of hyperopic defocus compensation in chicks exposed to short blue light (Nickla et al., 2023). Therefore, the effects of blue light exposure on myopia development and its underlying mechanism need to be further studied.

In this study, a longpass filter was used, which resulted in a 10% decrease in illumination. Therefore, to maintain an equal illumination level, a neutral density filter (ND 0.1) was used as a control. As shown in Figure 1C, the visible light transmittance of the ND and longpass filters was 81 and 84%, respectively. Notably, no significant differences were observed in pupil diameter between the two filters. However, slight constriction of the pupil (approximately 0.2 mm) after the defocus period (60 min) was found, which may be attributed to eye fatigue caused by prolonged viewing of a television placed 5 m away (McLaughlin et al., 2023).

Additionally, the dissociation that absence of axial shortening with significant choroidal thinning under the longpass filter—does not permit causal inference. Several alternative explanations exist. First, choroidal thickness has higher inherent fluctuation and may be more susceptible to measurement noise. Second, our 60-min defocus exposure may have been too short to induce a full response; a longer duration (Hussain et al., 2026) produces clearer defocus effects. Third, axial length and choroidal thickness may be regulated by partially independent pathways. Fourth, as noted in our limitations, the fellow eye control design (Hussain et al., 2026) can lead to conservative estimates due to interocular interactions, which could reduce the measured differences and contribute to the apparent dissociation. Future studies with longer durations or alternative designs (Wu et al., 2025) are needed to clarify these possibilities.

There are several limitations in this study. First, the participants were young adults, not children which emmetropization is more active. This is appropriate for a short-term mechanistic study, as adult exhibit robust and consistent defocus responses (Wang et al., 2016; Chiang et al., 2015; Read et al., 2010). Though our findings provide physiological insights into how short-wavelength light modulates acute axial responses in adults, but their clinical relevance for pediatrics myopia control remains unknown. Direct extrapolation to children would require further longitudinal studies. Second, ambient illuminance was set at 100 lux to simulate indoor lighting and maintain a photopic state, ensuring that short-wavelength-sensitive passway (S-cones and ipRGCs) are tonically active at baseline. While more pronounced defocus-driven choroidal changes frequently occur under dimmer conditions (~10 lux) (Read et al., 2018; Hoseini-Yazdi et al., 2020), that potentiate choroidal dynamic range. Our brighter condition may have attenuated the choroidal response. Nonetheless, the main finding that blue-light blocking abolishes axial shortening remains robust. Third, the use of the non-defocused fellow eye as the control condition, while common and practical in short-term defocus studies (Nickla et al., 2023), has an important methodological implication that deserves explicit emphasis. Increased evidence suggests that interocular interactions can occur, such that imposing defocus on one eye may produce a small, synergistic response in the fellow eye (Wu et al., 2025; Hung et al., 2018). consequently, the differences between the defocused eye and the control eye likely underestimate the true effect of the invention. In the other words, the reported changes in AL and ChT should be interpreted as conservative estimates, that the actual effects should be larger than observed. This design is a limitation when interpreting the absolute magnitude of the effects, although it does not affect the direction or statistical significance of the findings.

In summary, the present findings suggest that short-wavelength light contributes to the acute axial shortening response to myopic defocus under our experimental conditions, whereas its absence is associated with choroidal thinning instead. A critical question arising from these short-term observations is whether this pattern—axial stasis with choroidal thinning—persists or changes over longer time scales. The current cross-sectional design does not allow us to answer this question. Therefore, future investigations examining the long-term effects of specific spectral bands on ocular biometrics, particularly in children, will be essential to determine whether these acute findings have any relevance for myopia control strategies.

## Translational relevance

Light wavelengths and optical defocus may induce acute changes in ocular biometric parameters, which could be relevant to understanding mechanisms underlying myopia progression.

## Precis

Blocking short wavelengths attenuated the acute axial response to myopic defocus, suggesting that short-wavelength light may contribute to emmetropization and could inform future myopia control strategies.

## Data Availability

The raw data supporting the conclusions of this article will be made available by the authors on request.
